# Prosthetic failures in dental implant therapy

**DOI:** 10.1111/prd.12416

**Published:** 2022-02-01

**Authors:** Irena Sailer, Duygu Karasan, Ana Todorovic, Maria Ligoutsikou, Bjarni Elvar Pjetursson

**Affiliations:** ^1^ Division of Fixed Prosthodontics and Biomaterials University Clinics for Dental Medicine University of Geneva Geneva Switzerland; ^2^ Division of Prosthodontics Faculty of Dental Medicine University of Belgrade Belgrade Serbia; ^3^ Department of Reconstructive Dentistry Faculty of Odontology University of Iceland Reykjavik Iceland

**Keywords:** failure, implant restorations, mechanical, prosthetic complications, survival, technical

## Abstract

Both fixed and removable implant‐supported prostheses are well‐established methods for replacing missing teeth in partially or fully edentulous patients. Numerous systematic reviews have been performed in recent years to evaluate the survival and complication rates of implant‐retained fixed dental prostheses and implant‐retained overdentures, displaying high 5‐year survival rates ranging from 97.1% for fixed dental prostheses to 95%‐100% for implant‐retained overdentures. However, the survival rates only represent the prostheses remaining in use for a defined follow‐up time, and do not account for the potential prosthetic complications that may have arisen and influence the general success of the implant treatment. The most common technical complications of fixed implant‐retained single crowns are crown fracture, fractures of ceramic implant abutments, and esthetic problems. The predominant technical complication at multiple‐unit, implant‐retained fixed dental prostheses is fracture/chipping of the veneering ceramic. Reported technical complications for implant‐retained overdentures are overdenture fracture or chipping of the veneer materials, whereas mechanical complications include implant fracture, attachment failure, and attachment housing or insert complications. To reduce the risk of such failures, a comprehensive pretreatment diagnostic work‐up is essential, including defining the prosthetic goal with the aid of a wax‐up or set‐up and the associated ideal, prosthetically oriented three‐dimensional implant position. Furthermore, selection of the ideal type of prosthesis, including the respective implant components and materials, is important for clinical long‐term treatment success.

## 1 INTRODUCTION

Missing teeth can either be replaced by fixed or by removable implant‐supported prostheses. The clinical decision between the two differing types of restorations is based on anatomic, esthetic, and economic factors, and most importantly the wishes of the patient. High survival rates and low complication rates of the prostheses are an important prerequisite for the general success of treatment, as failures of the prosthesis may result in failures of the entire implant rehabilitation. One of the most important strategies to reduce the risk of failure is a comprehensive pretreatment diagnostic work‐up followed by the decision to fabricate either a fixed or a removable implant prosthesis. According to the prosthetic plan the number of implants should be defined, as well as their ideal three‐dimensional prosthetic positions in the mesio‐distal, bucco‐oral, and vertical dimensions.

In the case of single tooth gaps or partially edentulous areas framed by healthy neighboring teeth, fixed implant prostheses are usually indicated,^1^ and the decision‐making process is straightforward. In edentulous situations, however, the choice of fixed or removable implant prostheses is more complex. A major driver of the decision is facial esthetics (ie, the need for facial tissue support). If both fixed and removable prostheses may be considered, the next factor influencing the selection is the complexity of the surgical interventions required. With pronounced horizontal and/or vertical bone loss, large amounts of hard and soft tissue regeneration may be needed for fixed implant prostheses. Hence, in cases where there is a need for facial tissue support or large bone and/or soft tissue augmentations, removable implant‐retained prostheses such as implant‐retained overdentures are less invasive treatment options.^2^, ^3^

Numerous systematic reviews have been performed in recent years to evaluate the survival and complication rates of fixed and removable implant‐retained prostheses.^4^, ^5^, ^6^, ^7^, ^8^, ^9^ High 5‐year survival rates ranging from 97.1%^10^ for fixed to 95%‐100% for removable prostheses^4^ have been reported. In daily practice, however, high survival rates are not the only criterion defining the success of the implant treatment, as they only represent those prostheses remaining in use for a defined follow‐up time.^10^ They do not show whether or not these prostheses were affected by complications influencing the general success of the implant treatment.^10^ It is well known that patients experiencing complications with their fixed or removable implant prostheses are significantly less satisfied with the implant treatment than patients who do not experience problems.^11^

In a systematic review conducted by Salvi and Brägger^12^ as part of the proceedings of the 4th International Team for Implantology Consensus Conference, prosthetic risk factors were defined as either technical or mechanical risks. Therefore, based on this statement, the prosthetic complications can be considered as technical or mechanical complications. The technical complications represent those relevant to laboratory‐fabricated parts such as fracture and chipping of the veneering materials, whereas mechanical complications represent complications relevant to the prefabricated parts, such as implant fracture or abutment failures.^12^

As the reviews both fixed and removable implant prostheses exhibit different technical and/or mechanical problems over time.^4^, ^8^, ^10^, ^12^, ^13^

In the following, the most frequent prosthetic complications will be discussed for both fixed and removable implant prostheses, including risk factors for the complications, associations with early and late implant survival, and the prevention and management of complications. For simplification, the term “technical complications” will be used for both technical and mechanical problems.

## 2 IMPLANT‐SUPPORTED FIXED DENTAL PROSTHESES

Today, fixed implant‐supported crowns and multiple‐unit fixed dental prostheses can be fabricated out of metal ceramics or several dental ceramics. The selection of the material may have an influence on the outcomes.

### 2.1 Single implant‐retained crowns

Metal‐ceramic, single implant‐retained crowns were the gold standard for decades, yet today all‐ceramic implant crowns fabricated out of lithium disilicate or zirconia ceramics are successfully used as alternatives.^8^ In addition, leucite‐reinforced glass ceramics, alumina ceramics, or resin‐matrix ceramics can be considered for the fabrication of single implant‐retained crowns.^8^

The clinical outcomes for implant‐retained single crowns, as well as for their supporting implants, are very good. The overall 10‐year survival rate of implants supporting single crowns was demonstrated to be excellent at 95.2%, independent of which crown material was used.^5^ However, the overall 10‐year survival rate of the crowns was slightly lower at 89.4%.^5^ At the crown level, the survival rate was influenced by the materials used for their fabrication, as shown in a more recent review.^8^ The 5‐year survival rate of veneered alumina crowns was 96.8%, for veneered zirconia crowns it was 91.6%, while for monolithic lithium disilicate it was 91%. Hybrid resin‐matrix ceramic crowns only survived in 67%^8^ of cases. By comparison, metal‐ceramic, implant‐retained crowns exhibited a 5‐year survival rate of 98.3%.^14^

Overall, > 10% of crowns had to be replaced for different biologic or technical reasons in the first 10 years. The common technical complications for single implant‐retained crowns are fracture or loosening of the abutment/prosthetic screws, loss of retention of cemented crowns, and chipping or fracture of the veneering ceramic. The main reason for ceramic crown failure is complete fracture of the crown.^8^ Furthermore, fractures of ceramic implant abutments are considered as potential risk factors for the loss of implant‐retained crowns. Finally, esthetic problems may occur with the different restorative materials (ie, metals and ceramics), leading to a failure of the implant treatment.

#### 2.1.1 Fracture or loosening of retaining abutment/prosthetic screws

While abutment screw fracture is a rare complication, screw loosening was and still remains the most frequent technical problem with single implant‐retained crowns, with a cumulative 5‐year complication rate of 8.8%.^5^ Numerous developments of new screw designs and materials have led to a reduction of this problem over time of almost 50%. The 5‐year rate for screw loosening ranged from 3.9% to 26.2% in the literature published prior to 2000, and was 3.1%‐10.8% in studies published after 2000.^10^

Interestingly, crowns cemented to the supporting implant abutments suffered less from screw loosening than screw‐retained crowns^15^ Further evaluation of the literature showed that both the crown design (screw‐retainable or cementable) and the implant‐abutment connections (external or internal) have a significant influence on the risk of screw loosening.^10^

The stability of the screw joint can be influenced by the prosthetic implant axis. It has been shown that more screw loosening occurred with angulation‐correcting implants than with straight implants.^16^ Hence, the appropriate three‐dimensional position of the implant is a crucial parameter with screw‐retained implant prostheses to decrease the risk of complications. Furthermore, the number of retaining screws should be limited to one, as double screw systems exhibited a higher risk of screw loosening.^17^ In addition, manufacturer‐recommended torque values should be adhered to.^18^ Finally, implants with internal implant‐abutment connections are preferred to external connection systems, to reduce the risk of screw loosening.^19^

Today, implant‐retained crowns are more frequently screw‐retained than cemented,^15^, ^20^ following current recommendations to reduce the risk of peri‐mucositis and peri‐implantitis via excess cement.^20^ However, despite all these improvements, a long‐term stable solution eliminating screw loosening has not yet been found, and this risk has to be taken into consideration when treatment planning.

#### 2.1.2 Loss of crown retention

Loss of retention as a result of de‐cementation is the second most frequent complication with implant single crowns, occurring in 4.1% of cemented crowns after 5 years of function.^5^ The incidence reported in publications has decreased from 7.3% prior to 2000 to 3.1% after 2000.^10^ One possible reason for this improvement may be the more recent increase in the use of resin cements, indicated for the cementation of all‐ceramic crowns to the underlying titanium or zirconia/alumina abutments.

The restorative material plays an important role with incident technical problems. Metal‐ceramic crowns are not dependent on adhesive cementation to the substrate (the abutment) in order to receive sufficient strength for clinical function as they already have excellent material stability. For this reason, metal‐ceramic crowns are mainly cemented with conventional cements like zinc phosphate or glass‐ionomer cement. Ceramic crowns exhibit a reduced fracture strength compared with metal‐based crowns, and need to be chemically bound to the underlying substrate for improved clinical strength.^21^ Resin cements provide a chemical bond between the ceramic crowns and the underlying materials, thereby reinforcing the ceramic crowns. It has been shown that the 5‐year rate for loss of retention of ceramic crowns was only 1.1%,^8^ whereas for metal‐ceramic crowns the rate for loss of retention was five times higher at 5.5%, as reported in earlier reviews.^22^

The main disadvantage of resin cements is that they are very viscous, mostly translucent, and not radio‐opaque. In addition, they exhibit chemical bonding to the abutment substrate after curing. Hence, the removal of excess cement is significantly more difficult than with nonadhesive, opaque conventional cements.^23^

Research has shown that the position of the crown margin is an important influencing factor for remnants of excess cement: the deeper the crown margin then the larger the amount of excess cement.^24^ The removal of excess cement is difficult even at shallow crown margins.^15^, ^24^ During treatment planning for cemented implant crowns, the appropriate three‐dimensional position of the implant has to be carefully considered. To reduce the risk of complications associated with excess cement, screw retention of fixed implant prostheses is recommended.^15^, ^24^

#### 2.1.3 Chipping or fracture of the veneering material

Chipping of the veneering ceramic is the third most frequent complication with fixed implant prostheses. The rates reported range from 3.2% to 25.5%,^10^ with an overall 5‐year complication rate of 3.5%.^5^ Veneering ceramics are silica‐based ceramics with excellent esthetic properties; however, they have very low fracture strength values.^25^ They are applied to different metallic or ceramic framework materials, establishing a bond between the veneering ceramic and the framework material important for clinical performance.^25^ Several factors influence the risk of chipping of the veneering ceramic.

**FIGURE 1:**
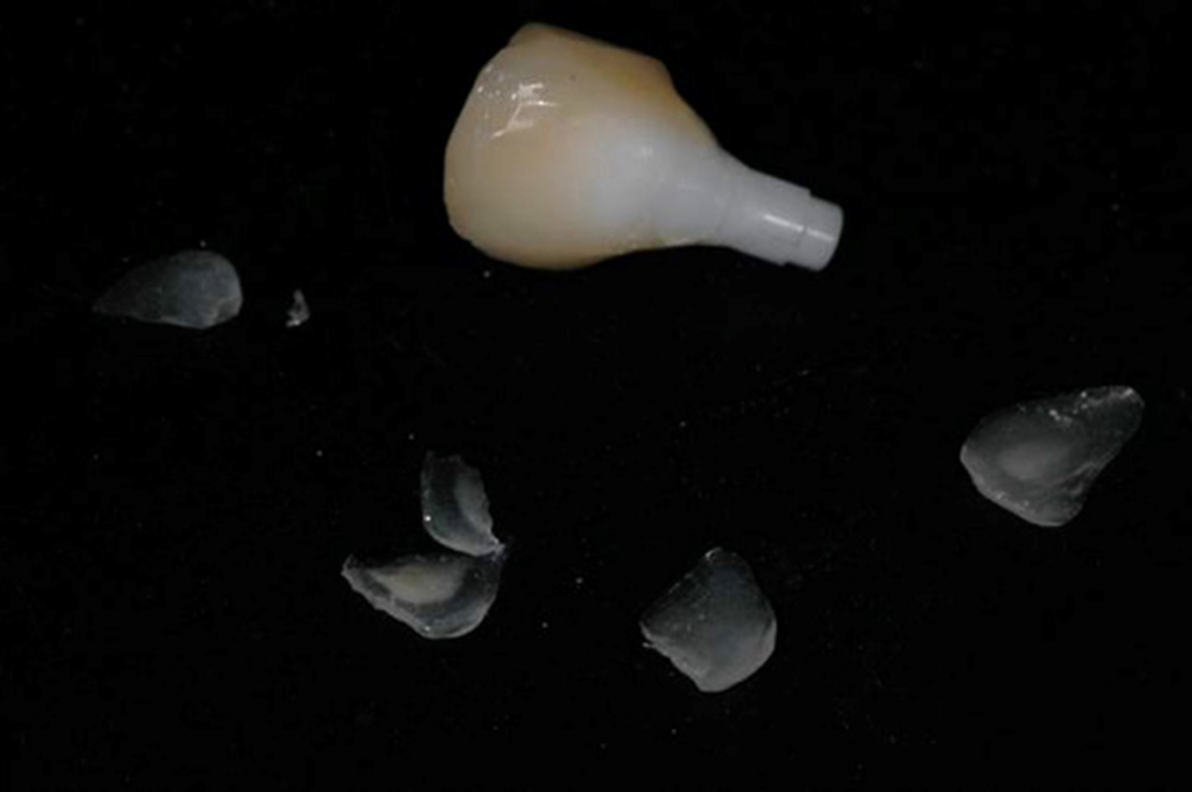
Multiple chipping of the veneering ceramic at a zirconia‐based implant single crown

The framework material plays an important role in preventing high chipping rates. It has been shown that veneered alumina or lithium disilicate crowns experienced chipping in 1.8% and 3.5% of cases after 5 years of function, respectively, whereas veneered zirconia crowns exhibited very high chipping rates of 11.8% over the same time frame.^8^ By comparison, the incidence of chipping in metal‐ceramic crowns was 3.5%^5^ (Figure 1).

Furthermore, the oral cavity is a very challenging environment for the performance of dental materials, most specifically for ceramics. Humidity, chemical attacks like acidic food or drinks, and changing temperatures lead to accelerated aging of ceramics.^25^ With aging, the risk of fracture or chipping increases. In addition, inherent defects and pores within the ceramic resulting from the manual veneering procedures^26^ further increase the risk of fracture or chipping. The long‐term integrity of the veneering ceramic is also influenced by the occlusion/function,^27^ as the forces applied to implant restorations are significantly higher than those applied to tooth‐borne restorations. It has been shown that the tactile sensitivity of dental implants is 8.7 times lower than that for natural teeth,^28^ hence the occlusal load on implant‐retained crowns is almost nine times higher than when supported by natural teeth.

Given the above, chipping of the veneering ceramic may not be avoided as a complication at veneered restorations. Therefore, current concepts may involve avoiding veneering materials by fabricating restorations out of monolithic ceramics. However, clinical studies on the monolithic lithium‐disilicate and zirconia ceramics are still scarce, and conclusions cannot yet be drawn. One review reported on a 5‐year cumulative survival rate of monolithic lithium‐disilicate implant crowns of 91%.^8^ No medium‐ to long‐term data on monolithic zirconia implant crowns are currently available.^29^ Therefore, it remains unknown whether or not monolithic implant crowns will have fewer problems with chipping. More research and development are needed before clinical recommendations on monolithic implant crowns can be made.

#### 2.1.4 Fractures of ceramic abutments

Fracture of ceramic abutments is a rare complication,^19^, ^30^ and the rates were 2.0% (95% confidence interval 0.5%‐7.4%) and 1.9% (95% confidence interval 0.7%‐4.8%) for internally connected ceramic abutments in the literature.^19^

The reviews demonstrated no differences in the survival rates of metallic and ceramic implant abutments for implants with external connections. Furthermore, no differences were found when comparing anterior and posterior regions,^30^ or internally and externally connected ceramic abutments.^19^ However, ceramic abutments exhibited more fractures than metallic abutments, a technical complication that inevitably leads to the failure of the implant restoration.^19^ Fracture of an internally connected ceramic abutment predominantly occurs in the internal part of the implant‐abutment connection,^31^ and in situations where the remnants cannot be removed from the internal connection, it may be necessary to remove the implant. For this reason, in internal connection implant systems, the application of ceramic abutments should only be recommended for the esthetic anterior regions.

Nowadays, the combination of internally connected titanium‐base abutments with zirconia abutments may serve as an alternative solution. A laboratory study showed significantly higher fracture strength values for titanium base‐supported zirconia abutments (hybrid solution) compared with externally or internally connected one‐piece zirconia abutments,^31^ and similar fracture strength values for zirconia abutments supported by titanium‐base abutments compared with customized titanium abutments.^32^ This new hybrid solution appears promising; however, clinical research remains scarce and no final clinical recommendations can be made at this stage.

#### 2.1.5 Esthetic complications

Esthetic problems can be a reason for the failure of implant treatment in specific clinical situations. A discoloration of the peri‐implant mucosa, caused by implant parts or components, can be a major problem with implants in the esthetic zone (ie, maxillary anterior and posterior regions in patients with a high smile line). Therefore, recent studies have focused on the effect of different restorative materials on the color of the peri‐implant soft tissues.

**FIGURE 2:**
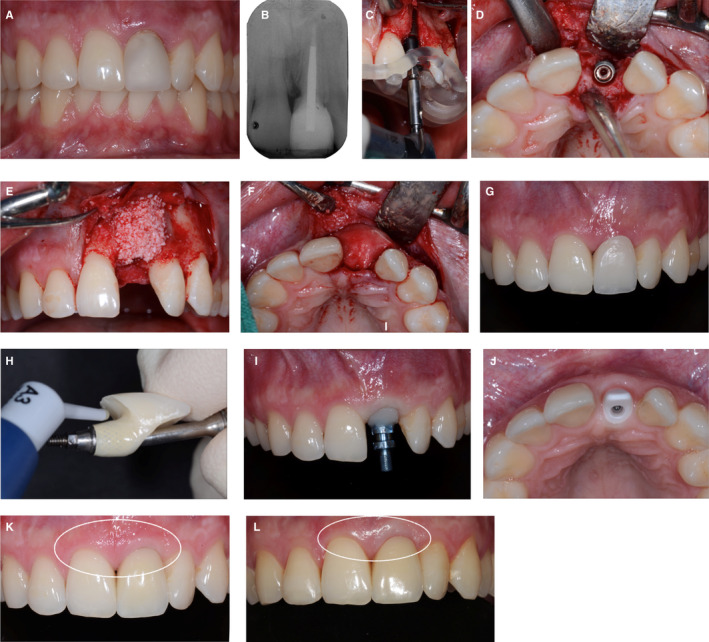
A and B, Clinical situation of a 24‐year‐old male patient with a failing left central incisor. The tooth lost vitality following an accident during the childhood of this patient. C and D, Replacement of the incisor by means of a bone‐level type of implant, with the aid of a guided surgery approach. E and F, Simultaneous guided bone regeneration to augment the volume of the ridge surrounding the implant with a xenograft and a collagen membrane (BioOss granules, BioGide membrane; Geistlich Pharma, Wolhusen, Switzerland). Submerged healing of the implant. G and H, Status after second stage surgery and insertion of a screw‐retained fixed implant provisional. Conditioning of the peri‐implant mucosa in a stepwise approach by application of a light‐curing resin (Tetric Flow, Ivoclar Vivadent, Schaan, Liechtenstein) to the submucosal part of the implant provisional, in order to receive a natural emergence profile of the implant restoration. I, Fixture‐level implant impression with a customized implant impression copying the submucosal part of the conditioned implant provisional for the final restoration. J, Fabrication and try‐in of the white zirconia abutment, foreseen for the support of a laboratory‐cemented glass‐ceramic crown, screw‐retained at delivery. K, Paleish, whitish discoloration of the peri‐implant mucosa at the implant in the left central incisor region. Status 30 min after insertion. L, Four‐year recall examination of the implant crown; note the still visible paleish appearance of the peri‐implant soft tissues, caused by the white zirconia substructure

It has been shown that metallic abutments and metal‐ceramic implant crowns caused a grayish discoloration of the mucosa in both laboratory and clinical studies.^33^, ^34^ The amount of discoloration and its effect on esthetic outcomes may be associated with the thickness of the mucosa.^34^ A critical soft tissue thickness of 2 mm was defined, with a grayish shine‐through of the metallic implant components in cases with thin soft tissues of < 2 mm.^34^ The color of tissues with thicknesses of > 2 mm was not influenced by the abutment or restorative materials. Hence, in esthetically important clinical situations, the clinical recommendation was to either use whiteish ceramic zirconia abutments and ceramic implant restorations in these cases, or to increase the thickness of the peri‐implant mucosa to values > 2 mm with soft tissue grafts.^34^ Interestingly, recent studies showed that the bright white color of zirconia also induces a soft tissue discoloration, leading to brightening and a pale appearance of the tissues (Figure 2A‐L).

As has been shown, discolorations at the level of peri‐implant soft tissues, as well as at the level of the implant restoration, can be perceived by both experts and laypersons,^35^, ^36^ therefore the esthetic outcome of implant restorations is key to their success. For this reason, several studies have focused on the ideal color of implant abutments and restorations. It has been shown that light pink or warm orange colors are more favorable than white.^37^, ^38^, ^39^

The influence of recent ceramic materials (ie, the colored and translucent lithium disilicate and zirconia ceramics) used for monolithic implant single‐unit and multiple‐unit fixed dental prostheses has yet to be investigated.

### 2.2 Multiple‐unit implant‐fixed dental prostheses

In contrast to single implant crowns, the selection of materials for multiple‐unit implant‐fixed dental prostheses is limited to metal ceramics and zirconia ceramics. For multiple‐unit fixed dental prostheses, zirconia displayed an inferior performance compared with metal ceramics, which are considered to be the gold standard.^40^, ^41^ In a recent review, metal‐ceramic, multiple‐unit, implant‐fixed dental prostheses exhibited cumulative survival rates of 98.7%.^42^ By comparison, zirconia‐ceramic, multiple‐unit, fixed dental prostheses had a significantly lower 5‐year survival rate of 93%.^42^ Another review reported better 5‐year cumulative survival rates for partial and full‐arch zirconia, multiple‐unit, implant‐fixed dental prostheses of 98.3% and 97.7%, respectively.^7^

In both reviews, the predominant technical complication was chipping of the veneering ceramic. Metal‐ceramic, fixed dental prostheses had a 5‐year chipping rate of 11.6%, while for zirconia‐ceramic, fixed dental prostheses the rate was 13.9%, with the difference being statistically significant.^42^

The predominant technical/mechanical complication at multiple‐unit, implant‐fixed dental prostheses is fracture/chipping of the veneering ceramic. Fracture of the ceramic framework and screw loosening are less frequent, but nevertheless are clinically relevant complications.

#### 2.2.1 Chipping of veneering ceramic

**FIGURE 3:**
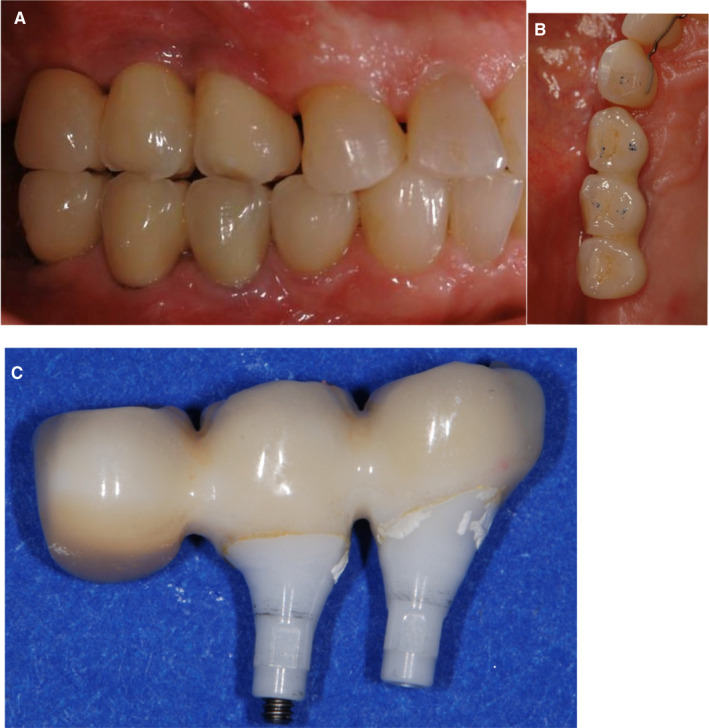
A‐C, Three‐unit cemented, zirconia ceramic‐fixed implant‐supported FDP (iFDP) exhibiting several prosthetic complications at the same time: multiple chippings of the veneering ceramic occurring at the buccal cusps only a short time after the insertion of the restoration because of inadequate occlusal design (ie, a cusp‐to‐cusp relationship of the maxillary and mandibular reconstructions in maximal intercuspidation). After the removal of the iFDP for the repair, remnants of excess resin cements were detected, which were associated with the reported difficulty of removal of excess cement at multiple‐unit cemented iFDPs

Chipping of the zirconia veneering ceramic was found in 34.8% of multiple‐unit, zirconia‐fixed dental prostheses in one review^7^ and in 50% of the fixed dental prostheses in another.^42^ Chipping of the veneering ceramic was reported for 8.8% of metal‐ceramic, implant‐fixed dental prostheses^22^ (Figure 3A‐C).

As with single‐unit, zirconia restorations, this problem remains unsolved, although the monolithic zirconia, implant‐fixed dental prostheses appear to offer a promising alternative.^43^, ^44^ Randomized controlled clinical trials with longer follow‐up periods are needed before clinical recommendations can be made.

#### 2.2.2 Fracture of zirconia frameworks

Fracture of zirconia frameworks was observed in 4.7% of restorations after 5 years of function, a complication that very seldom occurred with metal‐ceramic, multiple‐unit, fixed dental prostheses (0.2%).^42^ The extension of multiple‐unit, fixed dental prostheses is a crucial factor influencing the outcomes of zirconia as a framework material. Indeed, fractures only occurred with full‐arch, zirconia fixed dental prostheses; no fractures were observed with partially fixed dental prostheses.^7^

It has been shown previously that the size and the shape of the connectors are the most relevant parameters for the stability of multiple‐unit, zirconia fixed dental prostheses. The new types of monolithic translucent zirconia ceramics exhibit better esthetic properties than the previous yttria‐stabilized tetragonal zirconia polycrystal framework materials, yet lower strength values.^45^ For predictable outcomes, manufacturers’ recommendations need to be followed when designing these restorations.^45^

Unfortunately, no long‐term research is yet available for monolithic, multiple‐unit, zirconia fixed dental prostheses.

#### 2.2.3 Screw loosening

Screw loosening is a rare complication with both the metal‐ceramic and the zirconia‐ceramic, implant‐supported, multiple‐unit fixed dental prostheses.^7^, ^42^ Improvements in screw designs, screw materials, and torque values have led to a reported decrease in 5‐year screw‐loosening rates from 28.8% pre‐2000 to 4.7% post‐2000.^10^

## 3 IMPLANT‐RETAINED OVERDENTURES

implant‐retained overdentures are considered to be a favorable option to completely rehabilitate edentulous patients with a smaller number of implants less invasively, and in a more cost‐effective manner.^46^, ^47^ Furthermore, implant‐retained overdentures were reported to compromise patient satisfaction and masticatory efficiency less than fixed prostheses.^48^, ^49^, ^50^ High overall survival rates for implant‐retained overdentures have been reported for 5 years, ranging from 95% to 100%.^51^, ^52^ However, the survival rate should also be considered alongside complication rates to judge overall clinical success.

**FIGURE 4:**
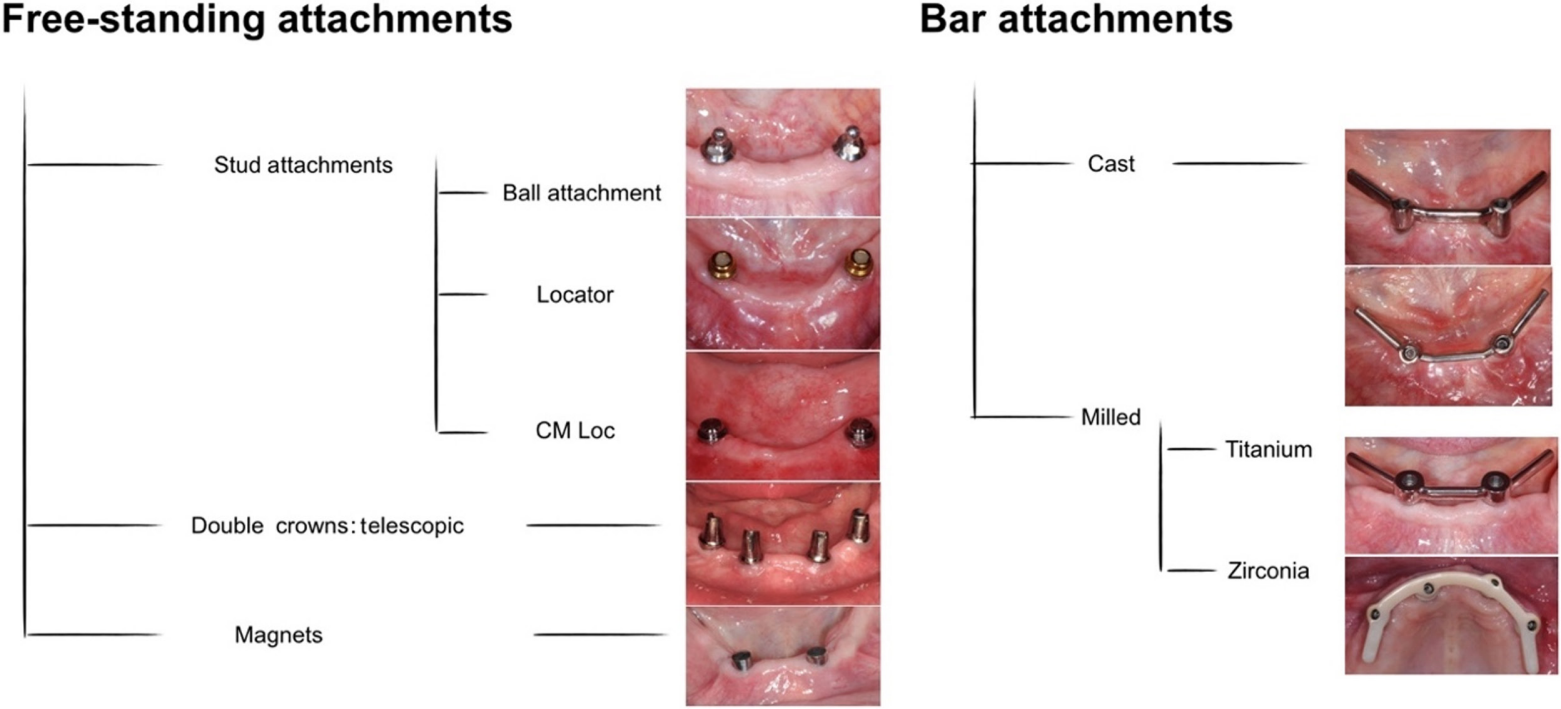
Summary of different implant‐retained overdenture attachment types

Various attachment systems provided by a large number of manufacturers are actively used to anchor overdentures to the underlying implants. The available attachment systems can be classified into two main groups, namely, free‐standing and splinted attachments (Figure 4). The most frequently used free‐standing attachments are stud attachments, such as ball, locator, CM loc, or telescopic attachments and magnets. Splinted attachments are also called bar attachments and can be divided into two groups, flexible and rigid bars. The most frequently used bar designs can be either cast or milled.^53^ In general, the attachment systems consist of a metal or plastic retainer (the female part or matrix) and an attachment part (the male part or patrix). While the matrix is embedded to the prosthesis, the patrix is attached to the implant.^54^

### 3.1 Technical complications and necessary maintenance

#### 3.1.1 Definitions

The technical complications of implant‐retained overdentures can include overdenture failure or chipping of the veneering materials, whereas the mechanical complications include implant fracture, attachment failure, and attachment housing or insert complications.^12^ Again, in the following, the term “technical complications” is used for both technical and mechanical problems. A list of possible complications with implant‐retained overdentures is provided in Table 1.

**TABLE 1:** Possible complications that may occur with implant‐retained overdentures

	Complication type	Definitions
Mechanical complications		
1	Patrix loose	Patrix component refers to stud attachments and/or its components as screws, as well as all inter‐abutment and cantilever bars/superstructures (round, ovoid, U‐shaped, milled, spark eroded)
2	Patrix activated	
3	Patrix replaced	
4	Patrix fractured	
5	Dislodged, worn, or loose matrix, or its respective housing	Matrix refers to O ring, resilient cap attachment, and magnets, as well as all types of metal alloy or plastic bar clips (single sleeve or multiple sleeve) or permanent resilient lining material connecting to inter‐abutment or cantilevered bars/superstructures
6	Matrix activated	
7	Matrix replaced	
8	Matrix fractured	
Technical complications		
9	Fractured implant overdenture	Puncture fracture of acrylic resin over patrix or fractured denture teeth
10	Reline of implant overdenture	
11	New implant overdenture reconstructed	

Walton et al^55^ reported a lack of a well‐defined protocols of evaluation for different implant‐supported restorations in order to be categorized as successful or unsuccessful over the long term. The authors stated the importance of anticipating the difference between regular prosthetic maintenance needs and prosthetic complications during the follow‐up period. Accordingly, the authors proposed a guideline to evaluate the implant restorations in academic or professional environments. In 2001, Payne et al^56^ elaborated upon and adapted this protocol, in particular for implant‐supported overdentures, encompassing:

1. prophylaxis, minor occlusal or anatomic corrections, polishing, asymptomatic, and peri‐implant/inter‐abutment mucosal enlargement not requiring excision.
2. prosthesis screw‐tightening or replacing not more than once a year after the first year.
3. Activation, repair, and replacement of either matrix or patrix within the limit of two replacements in the first year and a maximum of five replacements in 5 years.
4. Denture relining once in 5 years, considered as maintenance rather than a complication.

#### 3.1.2 Maintenance needs

A recent systematic review of mandibular implant‐retained overdentures^57^ reported that adjustments to the attachment system (activation, repair, replacement of patrix/matrix components) were the most common types of aftercare action, regardless of the attachment type. In 2012, Osman et al^58^ reported a similar result, concluding that adjustments and contouring of denture flanges followed a need for maintenance of patrices and matrices. These results should be interpreted with caution because differentiation of the events between ordinary maintenance needs and complications is not always well defined within the literature.

#### 3.1.3 Technical complication prevalence

##### Need of activation/loss/fracture of patrix or matrix retention component

The need for activation, replacement, or repositioning of a retention component, either the matrix or patrix, is the most frequently encountered event occurring with implant‐supported overdentures in both jaws.^57^, ^58^, ^59^, ^60^

“Time in function” is a more relevant factor than the attachment type. The incidence of a dislodged, worn, or loose matrix (or its housing) was more common after the first year with ball retainers, irrespective of the location of the implant overdenture. Nevertheless, the occurrence of other problems with attachments (eg, loosening or fractures) was not statistically different when the attachment types (ie, ball, bar, or magnet attachments) were compared for the first year of function and after 5 years.^59^

Furthermore, the rehabilitated jaw plays an important role. Andreiotelli et al^61^ reported that the ball and magnet groups presented more complications of the retentive elements (retention loss and wear, respectively) at mandibular implant‐retained overdentures. At the maxillary implant‐retained overdentures, supported by ≤ 4 implants, free‐standing designs exhibited a higher prosthetic failure rate than splinted implants, and maintenance was higher for solitary attachments, according to Sadowsky et al.^62^

Finally, an important factor in clinical situations that may compromise the retention of solitary anchors is the implant angulation. To reduce the incidence of patrix/matrix repairs, the use of ball, locator, and magnet attachment types may be indicated for an implant divergence of 10‐40 degrees.^60^, ^61^

There is a difference regarding the aftercare requirements between resilient (Dolder bars) and rigid (milled) bars. Fewer interventions for the retentive components were reported when using rigid anchorage from milled bars with metal reinforcement compared with resilient stabilization provided from round bars supported by four implants at maxillary overdentures.^57^, ^61^ This event is correlated clinically to the ability of the rigid anchors to resist movements and rotation of the overdenture, thus reducing the pace of wear of the attachments. Furthermore, a correlation between bar attachment type and fracture of distal extensions was reported. This complication is more common for the rigid bar group and is related to occlusal overload.^61^

##### Screw loosening/screw fracture/abutment loosening

In a review of the literature, Cehreli et al^59^ rated the frequency of screw or abutment loosening encountered at implant‐retained overdentures and found similar results for the different attachment types in both jaws. Controversially, Osman et al^58^ reported that screw loosening was the most common complication with maxillary implant‐supported overdentures with bar anchorage, occurring at a rate of 5% after 5 years of function. An increased inter‐implant distance may jeopardize the even stress distribution, resulting in more frequent abutment loosening.^58^ In addition, Assaf et al^57^ referred to a higher incidence of screw loosening for bar‐anchored mandibular implant‐supported overdentures compared with the ball‐retained group.

##### Fracture or replacement (fracture of acrylic resin, fractured denture tooth, fracture of framework or bar)/overdenture relining

The design of the implant overdenture, the location (jaw), and the time in function are relevant factors influencing the risk of technical complications. In the literature, maxillary implant‐retained overdentures presented a high rate of technical complications when designed without palatal coverage or without a metal reinforcement.^58^, ^61^, ^63^

Furthermore, Osman et al^58^ reported that acrylic resin and tooth fracture were more frequently encountered after 5 years of clinical function than at the beginning. This is in accordance with the reports of frequency of events, as rated by Cehreli et al,^59^ who stated that fractures, relines, and overdenture renewal were more frequently needed in a follow‐up period of > 5 years.

**FIGURE 5:**
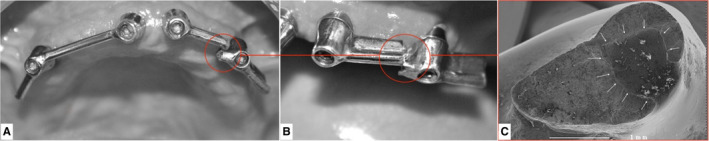
Clinical presentation of a patient with a fractured cast bar. A, Failure occurred after 10 months of clinical service; B, appearance of the fractured bar from the buccal aspect; C, Scanning electron microscope image of the fracture surface; a cave‐like casting defect is indicated by arrows. The mode of failure was detected as brittle overload fracture

Bar fractures are rare technical complications; however, in the case of a bar failure, renewal of the prosthesis may be required. According to a literature review there are six reported essential causes for metal framework fractures, including implant overdenture bars.^64^ These are inadequate metal thickness, poor solder joints, excessive cantilever length, alloys with inadequate strength, patients’ parafunctional habits, and improper framework design.^64^ Some of these are directly related to the bar itself, such as bar material, fabrication methods, or sensitivity (Figure 5).

Finally, the occlusal load and fabrication procedure have an impact on complications rates.^57^, ^58^, ^61^ The passive fit of prosthetic components and evenly distributed occlusal forces, without exceeding materials’ resistance and eliminating parafunctional load, reduce the incidence of problems during aftercare.

### 3.2 Risk factors for technical complications

To reduce the risk of prosthetic complications with implant‐retained overdentures, the selection of the attachment, the optimal number and location of implant fixtures, as well as consideration of the clinical factors, such as available prosthetic space and the opposing dentition, all need to be evaluated.

#### 3.2.1 Attachment type

**FIGURE 6:**
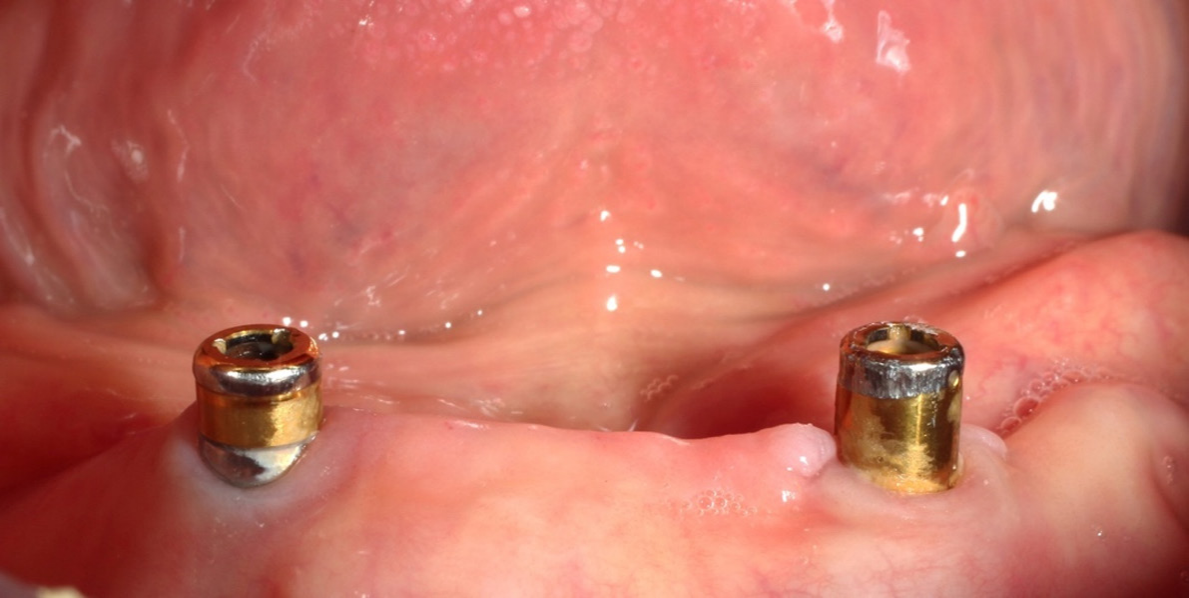
Extensive wear of locator attachment as a result of implant misalignment

Factors affecting the clinician’s preference with regard to attachment types can be variable. A recent global survey of 116 prosthodontists from 33 countries showed that the most commonly preferred attachment type was the locator attachment,^65^ and clinicians often made their selection based on subjective criteria such as their expertise, personal comfort, and their dental technician’s preference, or as influenced by marketing strategies.^66^ Nevertheless, each attachment system comes with its own clinical prerequisites and has different indications. Existing prosthetic space, inter‐implant distance, implant position and angulation, and number of implants are the main factors that should dictate the implant attachment of preference. Moreover, the maintenance requirements and complication rates arising can be related to such factors.^64^, ^67^ The consequence of ill‐positioned implants is, that the insertion path of the prosthesis and its fit will not be optimal, and this will result in a higher incidence of need for matrix change, or wearing of the patrix (Figure 6). In these cases, bar attachments are preferred to correct the axis deviations and achieve a better way of insertion.^68^ An incorrect selection of attachment will inevitably result in both higher maintenance requirements and complication rates.

The effect of attachment type on prosthetic maintenance and complication rates, as well as retention, stability, and patient satisfaction, has been studied in various clinical studies and reviews.^54^, ^59^, ^67^, ^69^, ^70^, ^71^

In a recent systematic review and meta‐analysis, Leao et al^70^ reported that there was no significant difference in prosthetic complications between splinted and free‐standing attachments. Although total complication rates did not differ, the observed complication types were different. With the bar attachments, fracture of the clip and overdenture were more common, whereas free‐standing attachments (like the ball attachments) demonstrated both a greater need for matrix change and deformation of the plastic components.^70^

In a comprehensive systematic review based on 49 studies, Cehreli et al^59^ evaluated the complications of implant‐retained overdentures for both maxillary and mandibular overdentures. The findings of this review showed that the attachment system had no effect on the incidence of prosthetic complications. A recent Cochrane review performed by Payne et al^71^ based on 294 mandibular overdentures reported insufficient evidence for the effectiveness of different attachment systems on prosthodontic success rates and prosthodontic maintenance. Hence, it was not possible to identify any preferred attachment system for mandibular overdentures. Compared with bar attachments, ball attachments showed a higher short‐term re‐treatment need because of repair of the attachment; however, it was stated that the quality of the evidence was low.^71^

A review conducted by Trakas et al^67^ stated that, independent of the attachment type (free‐standing or splinted), maintenance needs such as alteration of contour and repair of the matrix or patrix were high during the first year of clinical function.

Even although the reported data suggest that the difference in complication rates related to different attachments is negligible, it should be taken into consideration that the results are derived from systematic reviews. In these, the data were extracted from clinical studies that were executed in highly controlled clinical environments, and attachment‐type selection was made according to strict eligibility criteria. Therefore, it can be stated that as long as the attachments are used with the right indication, the expected complication rates are similar for all attachment types. The selection of the attachment type should be according to the above criteria (ie, existing prosthetic space, inter‐implant distance, implant position, angulation, and the number of implants).

#### 3.2.2 Number of supporting implants

The possibility of using two implants to support mandibular overdentures was first introduced by van Steenberghe et al (1987)^72^. Ever since then, mandibular overdentures have been extensively studied regarding the optimal number of supporting or retaining implants. Two consensus conferences concluded that a mandibular overdenture supported by two implants is the “gold standard” treatment for edentulous patients.^46^, ^73^ Because of their economic advantage and ability to improve patient satisfaction, overdentures supported by a single implant are also recommended by some authors.^74^, ^75^, ^76^ Nevertheless, it is necessary to evaluate the problems associated with the single implant overdenture treatment option, such as the risk of potential vascular damage and increased risk of implant overdenture fracture, because of the space occupied by the attachment housings and a decreased amount of acrylic resin.^77^ A meta‐analysis comparing single vs two implant‐retained mandibular overdentures, after 5 years in function, revealed that there were no significant differences regarding overall prosthetic complications in overdentures supported by a single implant compared with those supported by two implants.^78^ The most common failures reported were the replacement of attachment system components and fractures of the acrylic base, probably as a result of structural overload. Therefore, in single implant‐retained overdentures, the use of implants with a broad diameter, and reinforcing the denture bases with a metal framework, was recommended.

De Souza Batista et al^79^ also showed that there were no statistically significant differences in dental implant failure or prosthetic failure between overdentures retained by either one or two implants. Prosthetic repairs were mostly related to retention, such as the loss of retention from the retentive cap, and to denture‐base fractures. The most common complication was replacement of attachment system components and fractures of the acrylic base as a result of structural overload. Furthermore, Passia et al^80^ reported that the most frequent prosthetic maintenance intervention in single implant‐retained mandibular overdentures was activation or exchange of the matrix because of loss of retention.^80^

Even although there is consensus regarding the number of supporting implants for mandibular overdentures, the number of implants needed to support a maxillary overdenture remains controversial (Table 2). As reported by two recent systematic reviews conducted by Roccuzzo et al^81^ and Raghoebar et al,^51^ this question remains unanswered (Table 2).

**TABLE 2:** Selected reviews that report the optimal number of supporting implants for maxillary overdentures

Systematic review	Number of implants	Survival rate of maxillary overdentures
Roccuzzo et al (2012)^81^	Inconclusive	On the basis of available data, it is difficult to demonstrate that a particular number of implants offered a better outcome compared with another
Raghoebar et al (2014)^51^	Inconclusive	Maxillary overdentures supported by splinted implants have a high implant and overdenture survival rate (both > 95% per year) ≤ 4 nonsplinted implants; the overdenture survival rate is 98.8% per year ≥ 6 implants and a splinted anchorage; the survival rate is 99.5% per year ≤ 4 implants and a splinted anchorage; the survival rate is 96.9% per year
Di Francesco et al (2018)^4^	Not significantly influenced by the number of implants 94.7% to 100% for 6 or more implants with a splinted attachment 87.5% to 100% for 4 implants with a splinted attachment 95% to 100% for 4 implants without a splinted attachment 95% to 100% for fewer than 4 implants with or without splinted attachment	

Still, the literature indicates that mandibular overdentures can be supported by either one or two implants, whereas for maxillary overdentures, at least four supporting dental implants are recommended for good long‐term outcomes.

#### 3.2.3 Maxilla vs mandible

Both maxillary and mandibular overdentures were introduced to dental practice more than 30 years ago.^82^, ^83^, ^84^, ^85^ However, the number of patients with maxillary complete edentulism who seek implant therapy is lower than for mandibular edentulous patients, because of their greater satisfaction with complete dentures.^86^ Accordingly, patients edentulous in the maxilla who are willing to undergo implant therapy are more often the ones with compromised denture stability as a result of advanced bone resorption.^62^ Consequently, maxillary implants are more angulated facially and the teeth are arranged anterior and inferior to the residual ridge. This less than ideal tooth positioning, as well as anatomic differences, makes maxillary overdentures subject to unfavorable loads, resulting in lower survival rates and higher complication rates than for mandibular implant‐retained overdentures.

A systematic review performed by Bryant et al^87^ including 46 studies with a 5‐year follow‐up period reported maxillary and mandibular implant overdenture survival rates of 78%‐87% and 100%, respectively. Despite the fact that there was a difference between survival rates, similar maintenance event rates and numbers of visits for the 5‐year follow‐up period were reported. Watson et al^88^ reported three times higher fracture rates for overdentures in the maxilla compared with mandibular overdentures. Hutton et al^89^ reported a 27.6% rate of prosthetic failure of maxillary implant‐retained overdentures, which was nine times higher than for mandibular ones. A potential reason for these problems was the compromised bone status that led to higher bending moments at the terminal abutments of the maxillary implant‐retained overdentures, as reported by Jemt et al.^90^

An increased number of prosthetic complications was reported with maxillary implant‐retained overdentures without palatal coverage, therefore, palatal coverage is highly recommended, especially with a lower number of supporting implants.^59^, ^61^, ^91^

The maxillary masticatory mucosa is thicker than the mandibular mucosa, and the abutment heights are, accordingly, longer, leading to increased lever arms. This may be correlated to increased abutment‐related complication rates for maxillary implant‐retained overdentures compared with mandibular implant‐retained overdentures.^92^ Furthermore, bone characteristics, the shock‐absorbing properties, and hinge‐like shape of the mandible reduce the risk of force‐induced complications with mandibular implant‐retained overdentures.^93^

Overall, a higher incidence of technical problems has been detected in maxillary overdentures.^61^

#### 3.2.4 Available prosthetic space and opposing dentition

In general, more vertical and horizontal prosthetic space is required for the components supporting implant‐retained overdentures than for implant‐supported fixed dental prostheses. Where implant‐retained overdentures are considered as a treatment option, the jaws should accommodate enough space for the attachment, the housings/bar clips, and prosthesis thickness.^94^

Lack of sufficient prosthetic space will lead to inadequate dimensions of both attachments and prosthesis. However, because of limited existing data, direct correlation of the inter‐arch space with overdenture survival and success rates is not possible at present.^53^ Limited clinical evidence demonstrates that if the inter‐implant distance is < 8‐10 mm then the proper placement of bar clips is jeopardized and, accordingly, clip loosening occurs more frequently.^95^

Another potential risk factor for complications with implant‐retained overdentures is the opposing dentition. Complete edentulism has been shown to occur earlier and more frequently in the maxilla than in the mandible (40% vs 27%, respectively),^62^ and maxillary overdentures are opposed to a natural dentition more often than mandibular overdentures. It is difficult to find agreement in the literature regarding the effect of the opposing dentition on the complication rates of overdentures. Nevertheless, in a number of clinical studies evaluating maxillary implant‐retained overdentures, the opposing dentition seems to account for increased rates of complications or failure.^88^, ^96^, ^97^, ^98^, ^99^ The natural dentition can create higher forces and may lead to an increased need for maintenance and higher complication rates in opposing implant‐retained prostheses.

#### 3.2.5 Prosthetic material

A frequent technical complication in implant overdenture treatment is base fracture, hence, the design and materials play a crucial role in outcomes.^100^, ^101^

**FIGURE 7:**
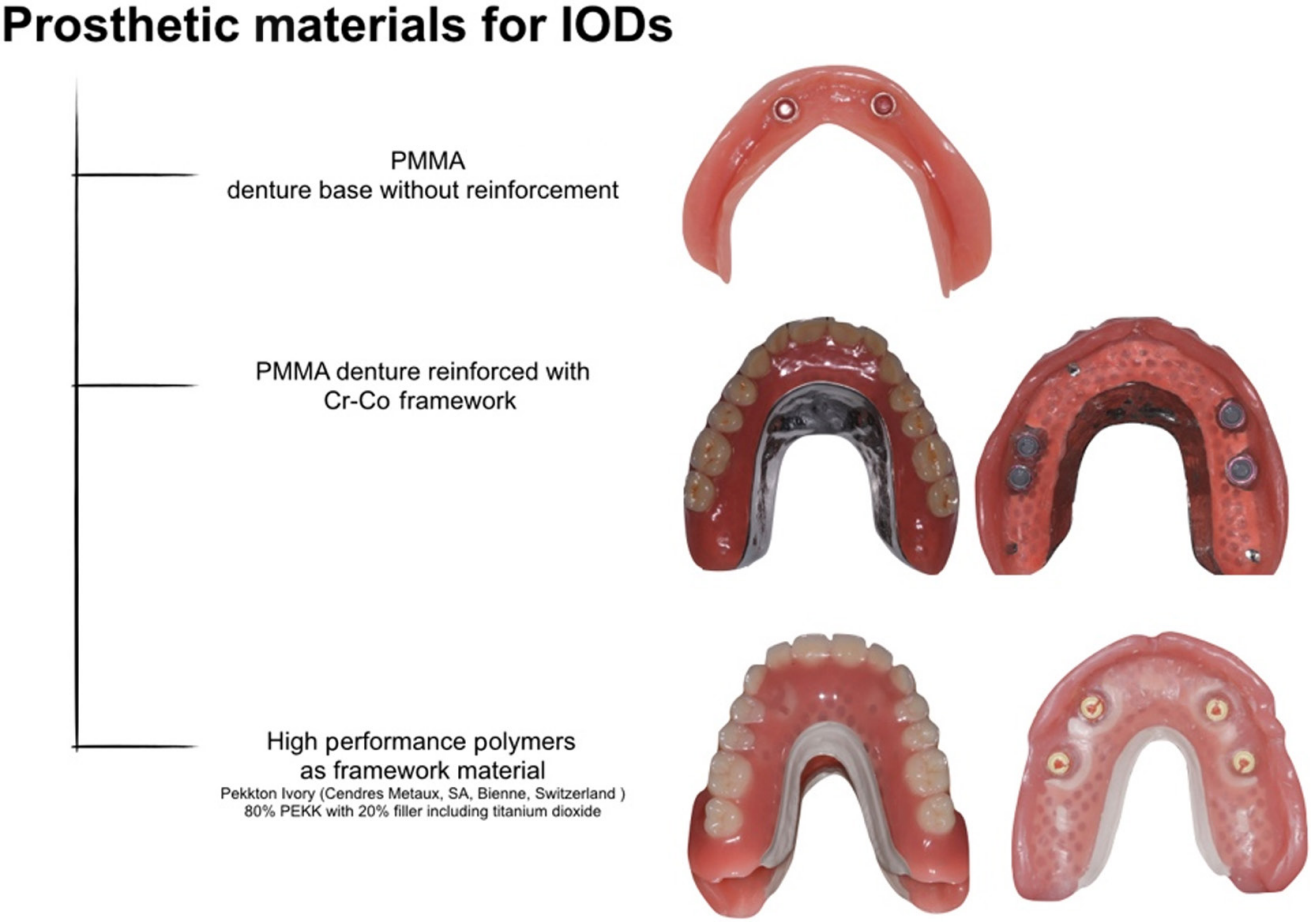
Types of prosthetic reinforcements for IODs. IODs, implant‐retained overdentures; PEKK, polyetherketoneketone; PMMA, polymethylmetharcylate

Denture‐base reinforcement is recommended to prevent technical complications of implant‐retained overdentures, because it improves the implant overdenture stiffness and decreases denture‐base deformation. Materials used for denture‐base reinforcement are metal, high performance polymers, and carbon and glass fibers (Figure 7).^102^, ^103^, ^104^, ^105^

It has been shown that reinforced implant‐retained overdentures exhibit a reduced risk for fracture compared with nonreinforced implant‐retained overdentures. The cobalt‐chromium framework is still the gold standard for framework fabrication today.^106^, ^107^, ^108^ However, nonmetallic framework materials like high performance polymers, such as polyetheretherketone, polyetherketoneketone, and glass fibers, are under investigation, as they may be beneficial because of their lower weight, better esthetics, and superior bonding ability to acrylic denture‐base materials.^109^, ^110^, ^111^ However, more data are needed before any recommendations can be made on these newer materials.

## 4 CONCLUSIONS

This review of the literature on fixed and removable implant‐retained prostheses demonstrates that technical complications cannot be avoided in either type of implant‐retained prosthesis. Technical complications can lead to the failure of implant treatment. To reduce the risk of this failure, a comprehensive pretreatment diagnostic work‐up, including defining the prosthetic goal with the aid of a wax‐up or set‐up and the associated ideal, prosthetic‐oriented three‐dimensional implant position, is crucial. Furthermore, selection of the ideal type of prosthesis, including the respective implant components and materials, is important for the clinical long‐term success of the reconstruction.
